# Editorial: Catch me if you can: cellular plasticity in tumor progression and drug resistance

**DOI:** 10.3389/fcell.2024.1470518

**Published:** 2024-08-15

**Authors:** Gian Luca Rampioni Vinciguerra, Ilenia Segatto, Julienne L. Carstens, Sara Lovisa

**Affiliations:** ^1^ Department of Clinical and Molecular Medicine, Faculty of Medicine and Psychology, Sant’Andrea Hospital, University of Rome “Sapienza”, Rome, Italy; ^2^ Unit of Molecular Oncology, Centro di Riferimento Oncologico di Aviano (CRO), IRCCS, National Cancer Institute, Aviano, Italy; ^3^ Department of Medicine, Division of Hematology and Oncology, University of Alabama at Birmingham, Birmingham, AL, United States; ^4^ O’Neal Comprehensive Cancer Center, University of Alabama at Birmingham, Birmingham, AL, United States; ^5^ Department of Biomedical Sciences, Humanitas University, Pieve Emanuele, Italy; ^6^ Department of Gastroenterology, IRCCS Humanitas Research Hospital, Rozzano, Italy

**Keywords:** cellular plasticity, epithelial-mesenchymal transition (EMT), drug resistance, tumor microenvironment (TME), metaplasia, cell stress adaptation, immune escape

Cellular plasticity is a broad term that encompasses the reversible mechanisms through which cells acquire different identities in response to homeostatic imbalances and cellular stress (Huyghe et al., 2024). In human malignancies, cellular plasticity is aberrantly activated at all stages of pre-malignant and malignant progression (Quintanal-Villalonga et al., 2020). This activation provides cancer cells with a dynamic capability to fluctuate across different states, allowing them to adapt to changing microenvironmental conditions and therapeutic pressures (Boumahdi and de Sauvage, 2020). Given its profound impact on sustaining tumor evolution, cellular plasticity is now recognized as a hallmark of cancer (Hanahan, 2022).

Over several decades of research, numerous insights have been gained into how plasticity promotes intratumor heterogeneity, immune evasion, drug resistance and metastatic dissemination in cancer. Despite these advances, the intricate molecular networks governing cellular plasticity remain only partially understood. Ongoing research continues to uncover novel molecular mechanisms and interactors that contribute to this process (Torborg et al., 2022). For example, accumulated evidence has highlighted the role of non-coding RNAs, epigenetic modifications, and signaling pathways in regulating cellular plasticity. These discoveries not only deepen our understanding of cancer biology but also identify new potential targets for therapeutic intervention (Mehta and Stanger, 2024).

This Research Topic represents a collective effort, aiming to gather and discuss the most recent findings that elucidate the role of cellular plasticity in tumor progression and evolution. By exploring the latest advances in this field, we seek to shed light on the complex interplay between cancer cells and the tumor microenvironment (TME), possibly identifying novel vulnerabilities for targeting this hallmark of cancer.

The article by Cammareri and Myant reviews the recent advancements and the transcriptomic mechanisms of metaplasia and epithelial-to-mesenchymal transition (EMT), two key examples of protective cellular plasticity in response to microenvironmental damage. Metaplasia, the adaptive process where one mature cell type converts into another, is discussed in the context of chronic inflammation of the esophagus and pancreas. The authors highlight its significant role in the development of epithelial dysplasia and the pathogenesis of esophageal and pancreatic adenocarcinoma. EMT, the process enabling cells to shift between epithelial and mesenchymal phenotypes (Haerinck et al., 2023), is also examined. The article summarizes recent findings and experimental models, discussing the intricate interaction between drug administration—an essential inducer of EMT—and how EMT in turn contributes to drug resistance

Metabolic plasticity has also emerged as a key mechanism promoting cancer cell adaptability to environmental stressors and therapeutic pressures (McGuirk et al., 2020). However, metabolic rewiring varies by tumor and therapy type, highlighting the need for systematic studies to develop effective therapies. To fill this gap, the review by Pendleton et al. explores the roles of permanent and plastic mitochondrial adaptations in therapy resistance. The article summarizes the experimental models of therapy-induced metabolic adaptation, emphasizing the clinical implications of targeting both permanent and plastic metabolic states. The authors highlight that oxidative phosphorylation in therapy-resistant cancer cells relies on diverse energy sources, underscoring that inhibiting these pathways is a promising strategy in preclinical studies. The review also discusses future directions and cautions for translating these findings into clinical practice, providing a valuable point of view for developing new therapeutic strategies.

Focusing on the specific interactors orchestrating the cellular plasticity, the review article by Liao et al. delves into the multifaceted role of LINK-A, a long non-coding RNA frequently dysregulated in human malignancies. The authors elucidate the molecular pathways regulated by LINK-A, particularly the HIF1α pathway, and how this novel interactor promotes various aspects of cellular plasticity, such as metabolic reprogramming, cell migration, invasion, and drug resistance. The review underscores the critical role of LINK-A expression in the interaction between cancer cells and cell populations within the TME, exploring mechanisms of immune evasion and suppression. Additionally, it discusses the potential translational significance of LINK-A expression, highlighting recent achievements in using LINK-A as a prognostic biomarker for clinical aggressiveness and poor prognosis, which can be evaluated in tumor samples and sera from patients with different tumor types.

The article by Lei et al. systematically explores the role of extracellular vesicles (EVs) in cancer cell plasticity and their contribution to the metastatic dissemination of gastric cancer. EVs are membrane-enclosed vesicles released by nearly all cell types, containing nucleic acids, proteins, and lipids that modulate the biological functions of recipient cells. It is not surprising that gastric cancer cells adopt this intercellular communication mechanism to reshape the surrounding environment into a pro-metastatic niche. The review examines the significance of gastric cancer-derived EVs in immunosuppression, angiogenesis, and phenotype switching within the TME. Additionally, the authors describe and discuss the role of EVs derived from cancer-associated fibroblasts and mesenchymal stem cells in promoting the metastatic ability of cancer cells, thereby underscoring the bidirectional role of EVs in tumor progression mechanisms.

The original investigation by Chong et al. explores the growth dynamics of breast cancer spheroids under varying extracellular matrix (ECM) conditions. Their findings highlight how variations in ECM pressure on cancer cells influence cell proliferation, induce apoptosis, and promote a switch to a mesenchymal phenotype, underscoring the critical role of matrix concentration and rigidity in modulating tumor expansion. A novel aspect of the study is the development of an algorithm to characterize the shear stress exerted by the expanding spheroids on the agarose matrix. The authors identified two distinct cell populations on the spheroid surface: one generating lower expansion forces, likely corresponding to non-dividing cells, and the other exerting higher forces, corresponding to dividing cells. The insights gained from this research are thoroughly discussed in the context of current literature and pave the way for developing more effective cancer treatments by targeting the biomechanical interactions within the tumor microenvironment.

The physical interaction between cancer cells and their microenvironment is further studied in the original report of Paxson et al. Recognizing that advanced prostate cancer extends beyond the fibro-muscular capsule of the organ, exposing cells to a contractile environment, the study investigates how mechanical stress fosters prostate cancer cell plasticity, possibly affecting sensitivity to therapeutics. Using a mouse-human tumor xenograft model, they isolated an aggressive, muscle-invasive cell population with distinct biophysical properties and a unique transcriptomic profile. High-throughput screening of several compounds revealed an altered profile of therapeutic vulnerability of muscle-invasive cells compared to controls. These findings suggest potential treatment strategies targeting the epigenetic landscape of aggressive tumors. Future research will focus on identifying effective combinations of epigenetic modifiers to eradicate early-stage metastatic cells.

Overall, articles in this Research Topic present a comprehensive overview of cancer cellular plasticity, providing novel insights and critical perspectives on its role in tumor evolution and treatment resistance. The collective findings underscore the importance of thoroughly understanding cellular plasticity to design effective cancer therapies. We extend our gratitude to the authors and reviewers for their dedicated contributions, and we hope the articles in this volume inspire future innovative research.

## References

[B1] Boumahdi S. de Sauvage F. J. (2020). The great escape: tumour cell plasticity in resistance to targeted therapy. Nat. Rev. Drug Discov. 19, 39–56. 10.1038/s41573-019-0044-1 31601994

[B2] Haerinck J. Goossens S. Berx G. (2023). The epithelial–mesenchymal plasticity landscape: principles of design and mechanisms of regulation. Nat. Rev. Genet. 24, 590–609. 10.1038/s41576-023-00601-0 37169858

[B3] Hanahan D. (2022). Hallmarks of cancer: new dimensions. Cancer Discov. 12, 31–46. 10.1158/2159-8290.CD-21-1059 35022204

[B4] Huyghe A. Trajkova A. Lavial F. (2024). Cellular plasticity in reprogramming, rejuvenation and tumorigenesis: a pioneer TF perspective. Trends Cell Biol. 34, 255–267. 10.1016/j.tcb.2023.07.013 37648593

[B5] McGuirk S. Audet-Delage Y. St-Pierre J. (2020). Metabolic fitness and plasticity in cancer progression. Trends Cancer 6, 49–61. 10.1016/j.trecan.2019.11.009 31952781

[B6] Mehta A. Stanger B. Z. (2024). Lineage plasticity: the new cancer hallmark on the block. Cancer Res. 84, 184–191. 10.1158/0008-5472.CAN-23-1067 37963209 PMC10841583

[B7] Quintanal-Villalonga Á. Chan J. M. Yu H. A. Pe’er D. Sawyers C. L. Sen T. (2020). Lineage plasticity in cancer: a shared pathway of therapeutic resistance. Nat. Rev. Clin. Oncol. 17, 360–371. 10.1038/s41571-020-0340-z 32152485 PMC7397755

[B8] Torborg S. R. Li Z. Chan J. E. Tammela T. (2022). Cellular and molecular mechanisms of plasticity in cancer. Trends Cancer 8, 735–746. 10.1016/j.trecan.2022.04.007 35618573 PMC9388572

